# Starvation Ketoacidosis: A Cause of Severe Anion Gap Metabolic Acidosis in Pregnancy

**DOI:** 10.1155/2014/906283

**Published:** 2014-05-20

**Authors:** Nupur Sinha, Sindhaghatta Venkatram, Gilda Diaz-Fuentes

**Affiliations:** Division of Pulmonary and Critical Care Medicine, Bronx Lebanon Hospital Center and Albert Einstein College of Medicine, Bronx, NY 10457, USA

## Abstract

Pregnancy is a diabetogenic state characterized by relative insulin resistance, enhanced lipolysis, elevated free fatty acids and increased ketogenesis. In this setting, short period of starvation can precipitate ketoacidosis. This sequence of events is recognized as “accelerated starvation.” Metabolic acidosis during pregnancy may have adverse impact on fetal neural development including impaired intelligence and fetal demise. Short periods of starvation during pregnancy may present as severe anion gap metabolic acidosis (AGMA). We present a 41-year-old female in her 32nd week of pregnancy, admitted with severe AGMA with pH 7.16, anion gap 31, and bicarbonate of 5 mg/dL with normal lactate levels. She was intubated and accepted to medical intensive care unit. Urine and serum acetone were positive. Evaluation for all causes of AGMA was negative. The diagnosis of starvation ketoacidosis was established in absence of other causes of AGMA. Intravenous fluids, dextrose, thiamine, and folic acid were administered with resolution of acidosis, early extubation, and subsequent normal delivery of a healthy baby at full term. Rapid reversal of acidosis and favorable outcome are achieved with early administration of dextrose containing fluids.

## 1. Introduction

A relative insulin deficient state has been well described in pregnancy. This is due to placentally derived hormones including glucagon, cortisol, and human placental lactogen which are increased in periods of stress [1]. The insulin resistance increases with gestational age and confers susceptibility to ketosis. Superimposed fasting and stress due to any cause may predispose to severe ketoacidosis. The exaggerated response to fasting with increased ketone formation in second trimester of pregnancy was first described by Felig and Lynch in 1970 [2].

Several studies suggest that the development of acidosis has an adverse maternal and fetal impact including fetal neurological impairment and fetal loss. Severe acidosis occurs rarely in pregnancy and is usually a result of diabetic ketoacidosis in women with type 1 diabetes mellitus, lactic acidosis secondary to sepsis, or rare conditions with metabolic consequences such as acute fatty liver of pregnancy. Similar metabolic changes are seen with poor dietary intake or prolonged fasting and resulting acidosis is referred to as “starvation ketoacidosis” [3]. Starvation ketoacidosis is encountered in many settings like prolonged fasting, malnutrition, gastric banding eating disorder, Atkins diet, and alcoholism, but seldom recognized, and is rarely a severe acidosis outside pregnancy [1, 4–7].

We report a very rare case of a pregnant patient presenting with acute severe starvation ketoacidosis after a period of vomiting, with favorable outcome after timely identification and intervention, stressing the importance of early recognition of this life-threatening entity as a cause of acute severe anion gap metabolic acidosis (AGMA).

## 2. Case Report

A 41-year-old female in her sixth pregnancy at 32nd week of gestation was admitted to the intensive care unit with history of recurrent vomiting for four days. Vomiting had worsened for 48 hours, last two episodes being bloody, prompting her to seek medical attention. She denied any fever, chills, abdominal pain, diarrhea, or urinary symptoms. She denied any past, personal, or family medical history and had no surgery in the past. Social history included occasional alcohol use which was continued during pregnancy. Urine toxicology screen was positive for cannabinoids. Patient was afebrile at the time of admission, appeared dehydrated, and was tachycardic and tachypneic, but normotensive. Initial laboratory (Table 1) revealed mild leukocytosis at 16,000 per cubic mm; severe AGMA with pH—7.162, PCO_2_—13, bicarbonate—5, and anion gap—31; and acute renal failure with creatinine—1.4 and normal lactate—1.4. Urine was positive for proteins and acetone. Acetone was present in moderate quantities in serum. Serum alcohol levels, acetylsalicylic acid, and acetaminophen levels were undetectable. Serum glucose level was 83 mg/dL and ammonia level 49 mcg/dL. Mild transaminitis was noted, with normal creatinine kinase (CK) levels. Patient required intubation and mechanical ventilation for worsening respiratory distress with Kussmaul's breathing and mild hematemesis. Esophagogastroduodenoscopy (EGD) revealed nonbleeding esophageal ulcerations. All septic work-up including blood, urine cultures, and chest roentgenogram were negative for infection. Initially, patient received normal saline, which was soon switched to 5% dextrose in lactated ringer mainly to avoid hyperchloremic acidosis. Thiamine and folic acid were given in view of prior alcohol use. Viability of fetus was confirmed by cardiotocographic tracings. Bicarbonate administration was considered but not given as acidosis resolved by 36 hrs and patient was successfully extubated on day 4 of hospital admission. Hydroxyproline levels, iron levels, ferritin levels, serum, and urine osmolarity were normal. Patient denied any use of antifreeze or any other toxic ingestion. A final diagnosis of starvation ketoacidosis due to recurrent vomiting with resulting poor oral intake, leading to severe AGMA and persistent hypokalemia, was made. Electrolytes were replaced and patient was monitored for refeeding syndrome. Patient was discharged with adequate follow-ups and delivered a healthy female baby by normal vaginal delivery at full term. The toxicology screen on the newborn was negative for cannabinoids. Her neurodevelopment is appropriate for age as noted in her most recent 18-month clinic visit.

## 3. Discussion

Metabolic acidosis of any etiology during pregnancy is concerning as it may lead to fetal lactic acidosis and hypoxemia [8]. Several studies have reported varying degree of adverse impact of maternal acidosis on neural development [8], including impaired intelligence [8–10] and fetal demise [11]. Identification of the underlying cause and timely intervention are of utmost importance in improving the fetal outcome.

In a patient presenting with increased AGMA, differential includes lactic acidosis, ketoacidosis (diabetic, starvation, or alcohol-associated), or toxic ingestions (methanol, ethylene glycol, and aspirin) [12].

Under normal circumstances with optimal glucose availability and normal insulin secretion, glycolysis generates pyruvate which enters the citric acid cycle for energy generation by the production of ATP. In any situation of low glucose availability, due to either starvation or insulin deficiency, there is deficiency of pyruvate entering the citric acid cycle due to depletion of glycogen stores. Alternative sources of energy in such circumstances are provided by generation of acetyl CoA from beta oxidation of fatty acids. The amount of acetyl CoA produced may exceed the capacity of the citric acid cycle, resulting in the generation of beta-hydroxybutyrate, acetoacetate, and acetone. The latter does not participate in energy generation and is either exhaled or excreted. Beta-hydroxybutyrate and acetoacetate are used as an alternative energy supply but their accumulation results in metabolic acidosis [1]. In an otherwise healthy individual, starvation requires at least 14 days to reach maximum severity, with mildly elevated ketoacids and pH usually being above 7.3 [1, 13]. Sufficient endogenous insulin secretion continues and prevents significant free fatty acid formation. Ketoacidosis is more severe in states of glycogen depletion, as seen in individuals with history of excess alcohol intake, and is augmented by lower insulin levels and perhaps higher levels of counterregulatory hormones as a result of volume depletion [1].

Normal pregnancy is characterized by relative insulin resistance, enhanced lipolysis, elevated levels of free fatty acids, and increased ketogenesis and even a short period of starvation may precipitate ketoacidosis [8]. Felig and Lynch first described the increased tendency to ketosis in pregnant women and demonstrated an exaggerated response to fasting during the second trimester, with increased ketone formation compared to nonpregnant women [2]. This concept was further clarified by Metzger et al., who demonstrated significantly higher levels of free fatty acids and beta-hydroxybutyrate after 12 hours of fasting in pregnant patients in their third trimester compared to the nonpregnant group [14]. This exaggerated response in late pregnancy is described as “accelerated starvation” and is possibly a mechanism for rapidly adapting the mother to the metabolism of fat so that less expendable fuels such as glucose and amino acids can be spared for the growth of the fetus. It is postulated that the predisposition to ketosis is the result of a relative lack of insulin, augmented by the presence of placentally derived counterregulatory hormones such as glucagon, cortisol, and human placental lactogen. These hormones are increased during periods of stress such as vomiting [1].

Predisposition to acidosis is further augmented in late pregnancy by state of respiratory alkalosis due to central respiratory stimulation by progesterone, lung volume changes, and altered compliance leading to hyperventilation and a reduction in PCO_2_ [15, 16]. The chronic respiratory alkalosis is compensated by renal bicarbonate excretion, resulting in lower plasma bicarbonate concentration and reduced buffering capacity [15, 17], thus contributing to increased ketotic tendency in third trimester pregnancy [15, 18].

In our case, bicarbonate level of 5 was remarkable as vomiting leads to metabolic alkalosis secondary to loss of hydrochloride. Serum lactate levels were normal which made the diagnosis of lactic acidosis unlikely. Diabetic ketoacidosis was ruled out by normal glucose levels, absence of prior history of diabetes, and normal HbA1C. Additionally, she has remained nondiabetic during future follow-ups with us, thus ruling out the possibility of euglycemic diabetic ketoacidosis. She denied any history of eating disorders, including anorexia, or being on any specific diet. Patient denied any toxic ingestion or recent alcohol intake. Serum salicylate, alcohol, and acetaminophen levels were negative. Extensive search of literature did not reveal association of cannabinoids with severe ketoacidosis [19–21]. The rare differential of transient oxoprolinuria [22] was considered but hydroxyproline levels and ammonia levels were normal. In view of mild transaminitis and low platelets, HELLP syndrome was considered but discarded in absence of hemolysis and thrombocytopenia. Her normotensive status, absence of history of hypertension, and only mild proteinuria ruled out the diagnosis of preeclampsia. Starvation ketoacidosis was the most plausible diagnosis. We hypothesize that accumulation of ketones in this woman with recurrent vomiting and poor intake of 4-day duration in her third trimester pregnancy, augmented by underlying state of depleted glycogen stores in the setting of history of alcohol intake, may have resulted in profound starvation and resulting acidosis. The rapid resolution of the AGMA following administration of dextrose containing intravenous fluids supports our diagnosis.

Starvation ketosis in pregnancy has been described in the literature [1, 3, 11, 15, 18, 23–25], but the few cases presenting with such severe acidosis have been associated with diabetes mellitus. Recently a single case of a pregnant patient with severe acidosis was reported in association with muscular dystrophy [26]. Almost all reported cases required emergent Caesarian section in addition to dextrose administration. Only one reported case [3] resolved with administration of 10% dextrose.

This case is unique on account of the extreme severity of ketoacidosis in the setting of acute starvation, absence of comorbidity, and the favorable fetal and maternal outcome with early administration of dextrose containing fluids.

## 4. Conclusion

Starvation ketoacidosis in pregnancy may present as severe anion gap metabolic acidosis after short period of acute starvation. Third trimester pregnancies are prone to ketosis and starvation ketoacidosis may develop rapidly in the presence of inadequate caloric intake. The mother may appear well despite the severity of metabolic derangement, which can help differentiate this from other causes of raised anion gap metabolic acidosis.

Early recognition of the pregnant patient at risk for starvation ketoacidosis is essential for fetal outcome. Late recognition and thereby delay in treatment are associated with a greater risk for impaired neurodevelopment.

Intensivist and obstetricians should be aware of this entity especially in a pregnant patient with concurrent history of alcohol use. We recommend timely provision of carbohydrates in cases of suspected starvation in pregnancy; this allows endogenous insulin secretion and inhibits further ketone production. In patients with marginal insulin reserves, glucose levels should be monitored closely and introduction of insulin along with dextrose may be considered as part of the treatment.

## Figures and Tables

**Table 1:** Laboratory parameters.

	At admission	12 hours	24 hours	48 hours
pH	7.158	7.199	7.248	7.403
pCO_2_ (mmHg)	19.0	8.8	20.6	24.1
pO_2_ (mmHg)	29.4 (venous)	135	246	187
HCO_3_^−^ (mg/dL)	5	4	10	16
Anion gap (mmoles/L)	31	29	16	12
Acetone	Moderate	Moderate	Negative	Negative
Lactate (mmoles/L)	1.4	1.0	1.0	1.0
Glucose (mg/dL)	83	80	110	155
Creatinine (mg/dL)	1.4	1.4	1.1	1.2
Potassium (mEq/L)	5.5	3.7	3.1	3.0
Hemoglobin (g/dL)	14.2	12.7	10.1	9.2
Albumin (g/dL)	4.1	—	3.1	3.1
AST (U/L)	37	—	29	34
ALT (U/L)	41	—	34	35
Alkaline phosphatase (U/L)	152	—	116	115
Bilirubin (mg/dL)	0.2	—	0.3	0.3
Serum osmolality (mOsm/Kg)	309	—	—	283
Urine osmolality (mOsm/Kg)	632	—	—	—
Urine analysis	Ketones 2+	—	—	—

## Abbreviations

AGMA: Anion gap metabolic acidosis
CK: Creatinine kinase
EGD: Esophagogastroduodenoscopy
HELLP: Hemolysis, elevated liver enzymes, and low platelets.
